# Protein and Leucine Requirements for Maximal Muscular Development and Athletic Performance Are Achieved with Completely Plant-Based Diets Modeled to Meet Energy Needs in Adult Male Rugby Players

**DOI:** 10.3390/sports12070186

**Published:** 2024-07-08

**Authors:** David M. Goldman, Cassandra B. Warbeck, Micaela C. Karlsen

**Affiliations:** 1 Department of Public Health, University of Helsinki, 00014 Helsinki, Finland; 2 Department of Research and Development, Metabite Inc., New York, NY 10036, USA; 3 Department of Family Medicine, University of Alberta, Edmonton, AB T6G 2R3, Canada; cwarbeck@ualberta.ca; 4 Department of Research, American College of Lifestyle Medicine, Chesterfield, MO 63006, USA; mkarlsen@lifestylemedicine.org; 5 Departments of Applied Nutrition and Global Public Health, University of New England, Biddeford, ME 04005, USA

**Keywords:** rugby, athletic performance, muscle mass, sports nutrition, plant-based, protein, leucine

## Abstract

Rugby athletes consume large amounts of animal protein in accordance with conventional dietary guidance to increase muscle mass and strength. This misaligns with national dietary guidelines, which suggest limiting meat consumption for chronic disease prevention. The ability of completely plant-based diets to satisfy the nutritional needs of rugby players has not been explored. This study scaled nutrient data from a large population consuming completely plant-based diets with limited supplemental protein to meet the calorie requirements of adult male rugby athletes to assess whether protein and leucine recommendations for muscular development and athletic performance would be achieved. Calorie requirements were estimated from research that employed the doubly labeled water method, and dietary data from the Adventist Health Study-2 were scaled to this level. The modeled protein level was 1.68 g/kg/day, which meets recommendations for maximal gains in muscle mass, strength, and athletic performance. The modeled leucine level was 2.9 g/meal for four daily meals, which exceeds the threshold proposed to maximally stimulate muscle protein synthesis in young men. These results indicate that consuming large portions of completely plant-based meals can satisfy protein and leucine requirements for maximal muscular development and athletic performance in adult male rugby athletes while aligning with public health recommendations.

## 1. Introduction

Protein intake is a focal point in the diets of rugby players [1]. The most frequent dietary advice these athletes receive regards protein, which is also the nutritional domain where rugby players have demonstrated the largest deficit in knowledge [2]. The primary reason that protein is reportedly consumed by rugby players is to increase muscle mass and strength [3], and more than one-third of rugby players report believing that the more protein one eats, the more muscle they build [2]. The fact that muscles do not derive most of their energy from protein was correctly reported by a minority of rugby players [2]. Accordingly, rugby players prioritize protein-based foods to increase energy and promote fueling [1].

The International Society of Sports Nutrition recommends animal-sourced protein for the enhancement of muscle mass [4], which has been shown to predict physical performance in rugby players [5,6]. Meal plans created for professional male rugby league players also prioritize protein from animal sources [7]. This advice is evident in the eating patterns of rugby players, for whom the majority of protein choices are animal-based and consist largely of beef, chicken, dairy, and eggs [1,2,8,9,10]. This conflicts with national public health guidelines in the USA, England, and Australia to reduce the intake of red and processed meat [11,12,13] as well as international recommendations to decrease meat intake and increase the consumption of plant foods [14,15]. Diets that include larger quantities of animal-sourced protein are associated with increased risk of incident cardiovascular diseases (CVDs) [16,17] and CVD mortality [18,19], the primary cause of death among former rugby players, followed by cancers, neurodegenerative diseases, and respiratory diseases [20]. Although some research has found favorable lipid profiles in rugby players compared to non-athlete controls [21], other studies have reported equivocal findings [22] or a high prevalence of atherogenic lipid profiles and other risk factors for CVD [23,24,25]. The majority of male professional rugby players have one or more CVD risk factors [26], and a significantly higher incidence of hypertension and diabetes has been reported in rugby players compared to the general population [25]. Recommendations have therefore been made that rugby players engage in preventative strategies to mitigate their elevated CVD risk [25].

The Academy of Nutrition and Dietetics (AND) recommends vegetarian and vegan diets for CVD prevention and treatment, and affirms that they are also appropriate for athletes [27]. Eight percent of athletes report following these dietary regimens [28], which can be categorized as plant-based diets when they emphasize the intake of fruits, vegetables, whole grains, legumes, nuts, seeds, and plant products and reduce or eliminate animal-based food consumption [29]. The research team has previously defined a “completely plant-based diet” as a diet that consists mainly of whole, plant foods and is bereft of animal products, demonstrated by fiber intakes that satisfy recommendations [30,31]. Recommendations from the AND diverge from the mainstream ideology that plant-based diets are disadvantageous for muscular development and athletic performance, which is often attributed to insufficient protein quantity and quality, including relatively low contents of dietary leucine [4,32,33,34,35,36,37,38,39]. Leucine may play a particularly significant role in the development of muscle mass and strength due to its concomitant ability to transport other amino acids into muscle tissue and effectively stimulate muscle protein synthesis (MPS), an anabolic process that is essential to muscle growth [40]. Other limiting amino acids within plant-based proteins that have been proposed to contribute to inferior amino acid compositions relative to animal-based proteins include isoleucine, lysine, methionine, threonine, and tryptophan [41]. However, the levels of each of these amino acids satisfy requirements in virtually all adults in a Western population following plant-based diets who meet dietary protein needs, particularly when legumes, nuts, and seeds are substituted for animal protein [42]. Nonetheless, athletes who follow plant-based diets are advised to meticulously plan and monitor their diets to ensure adequate intakes of protein, specific amino acids, and other key nutrients [37,41,43,44,45,46]. Previous investigations comparing the effects of resistance training (RET) and well-planned, isonitrogenous omnivorous versus completely plant-based diets on muscle mass and strength outcomes have not demonstrated significant differences between conditions [47,48]. However, protein supplements supplied a large proportion of the protein consumed in these studies, precluding an assessment of the degree to which completely plant-based diets that comprise mostly whole foods and include limited protein supplementation can meet the elevated protein needs of athletes pursuing maximal muscle mass and strength for their sport.

It has been theorized that plant-based diets may be suitable for rugby players [1,49]. However, to the authors’ knowledge, data pertaining to the nutrient intakes of rugby players following completely plant-based diets have not been published. This research team has modeled dietary values in other competitive athletes requiring high levels of muscular strength and hypertrophy [30,31], but investigations specific to rugby players are absent. Therefore, the objective of this study is to assess whether completely plant-based diets, modeled to satisfy the caloric demands of rugby players, can promote athletic performance and health by supplying enough protein and leucine to support maximal gains in muscular strength and hypertrophy while fulfilling micronutrient requirements.

## 2. Materials and Methods

This analysis follows the previously described methods that have been used to model protein and leucine levels in competitive male bodybuilders and American football players [30,31]. In brief, dietary values were obtained from the Adventist Health Study-2 (AHS-2) because this study surveyed the largest cohort that followed a plant-based (vegan) diet (*n* = 5694) and exceeded dietary fiber requirements (47 versus 19–38 g/day) [50,51]. Energy requirements were obtained from a recent systematic review of total energy expenditure (TEE) in collision team-sport athletes using doubly labeled water [52], which is considered the gold standard for TEE assessment on account of its accuracy [53]. The mean TEE in adult rugby players in the systematic review was 4587.5 kcal/day [52], and this value was used for energy requirements in this modeling study. Adults included in the study were U24 Elite English Rugby players competing in the Championship Rugby Union or professional Super League, with a mean age of 23 years and mean body mass of 99 kg, and throughout the measurement period, lasting 14 days, training and match details included 1–9 light training days (defined as including one resistance or rugby training session), 1–4 heavy training days (defined as including both resistance and rugby training sessions), 0–2 rugby matches, and 4–9 rest days [52].

Dietary protein requirements were determined by consulting publications from the International Olympic Committee (IOC). IOC resources have been used to establish reference ranges for protein intake in rugby athletes [54], and they state that optimal protein intakes for strength-training athletes are 1.6 g/kg/day [55]. This level resides within the range of protein intake recommendations for rugby athletes reported elsewhere in the scientific literature [7,22,56,57]. Protein requirements for athletes are determined relative to body mass [58]. Body mass values in adult rugby players were obtained from the systematic review from which athlete energy requirements were determined [52]. The body mass range for this demographic was 98.3–99.4 kg, and the midpoint was used to calculate protein requirements. The product of the body mass midpoint (98.9 kg) and the recommended protein intake (1.6 g/kg/day) output the mean protein requirement for rugby athletes to maximize gains in muscle mass, strength, and athletic performance. Mean protein intake in the AHS-2 cohort adhering to a vegan diet was reportedly 14.5% of total calories [50]. The product of this percentage and the TEE value yielded the absolute protein level (g/day) modeled in rugby players. Dividing this value by mean body mass output the mean relative protein level (g/kg/day).

The procedures for determining leucine requirements were detailed previously [30,31]. In alignment with these methods, a target was set at 2 g of leucine per meal for four meals daily, amounting to ≥8 g per day. The mean leucine contents of commonly consumed animal and plant proteins are 8.8% and 7.1%, respectively [59], and leucine contents were determined as the product of the value for plant proteins and total dietary protein.

Micronutrient and other key nutrient modeling methods were described previously [30,31]. The Dietary Reference Intakes (DRIs) presented in the United States Department of Agriculture Dietary Guidelines for Americans (USDA DGA) 2020–2025 were used to determine target values for omega-3 fatty acids, linoleic acid, saturated fat, fiber, and vitamins A, B6, B9, B12, C, D, E, as well as calcium, iron, magnesium, phosphorus, potassium, sodium, and zinc [11]. Levels pertaining to men 19–30 years of age were chosen as targets because these parameters characterize the demographics of elite rugby players [5,60]. Government dietary recommendations from Public Health England (PHE), the National Health Service (NHS), the European Food Safety Authority (EFSA), the National Health and Medical Research Council of the Australian Government Department of Health and Ageing, and the New Zealand Ministry of Health were also used as nutrient targets to facilitate application to rugby athletes in other countries [61,62,63,64,65].

## 3. Results

### 3.1. Protein Requirements and Levels

**Figure 1:**
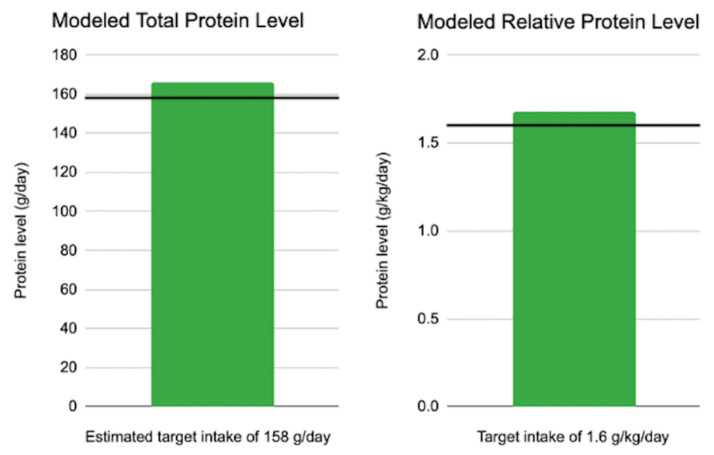
Protein and leucine values scaled to meet the calorie needs of adult male rugby athletes following completely plant-based diets, relative to targets proposed to maximize athletic performance. g: grams; kg: kilograms.

Mean protein requirements for rugby players were calculated to be 158 g/day, as shown in Table 1. The absolute protein level modeled in rugby players was 166 g/day of protein. Expressed relative to body mass, this amounts to a daily protein level of 1.68 g/kg/day, which meets the recommendations of 1.6 g/kg/day, as shown in Figure 1.

### 3.2. Leucine Levels

**Figure 2:**
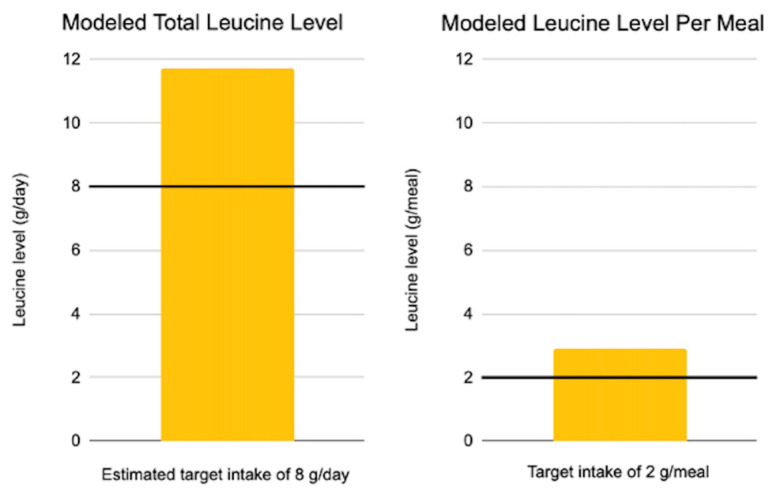
Leucine values scaled to meet the calorie needs of adult male rugby athletes following completely plant-based diets, relative to targets proposed to maximize muscle protein synthesis. g: grams.

Modeling indicated leucine levels of 11.7 g/day, or 2.9 g/meal at each of four daily meals. These values reached the targets, as presented in Figure 2.

### 3.3. Micronutrient and Other Nutrient Levels

The diets exceeded national targets set in the USA, England, Australia, and New Zealand for omega-3 fatty acids (including total omega-3 fatty acids and alpha-linolenic acid), linoleic acid, fiber, calcium, iron, magnesium, phosphorus, potassium, zinc, and vitamins A, B6, B9, B12, C, and E. Vitamin D values met the recommendations in Australia and New Zealand (578 vs. 200 IU/day) and approached, but did not meet, the recommendations in the USA and England (600 IU/day). Mean sodium values surpassed recommendations (8099 vs. 2000–2300 mg/day) and mean saturated fat levels (5% of calories) remained below recommended upper limits (<10% of calories). The results are presented in Table 2.

## 4. Discussion

This investigation assessed the suitability of completely plant-based diets, adjusted to meet the energy demands of adult male rugby players, in attaining the recommended quantities of protein, leucine, and micronutrients essential for supporting athletic performance, muscular development, and overall nutritional requirements. The results suggest that these modeled diets satisfy protein requirements for the maximization of athletic performance and gains in muscle mass and strength (1.6 g/kg/day) in rugby athletes by containing 1.68 g/kg/day protein [7,22,54,55,56,57]. Additionally, the results suggest that scaled, completely plant-based diets meet leucine needs for the maximal stimulation of MPS (2 g/meal) [67,68,69], providing 11.7 g/day or 2.9 g/meal at four daily meals.

The outcomes of this investigation may have significant implications for public health and athletic domains. They suggest the prospect of harmonizing guidelines aimed at the prevention and treatment of chronic diseases with efforts to enhance muscle mass and strength, as well as athletic performance, in rugby players, who experience significant risk of CVD. This modeling study indicates that rugby players can meet their dietary requirements for peak athletic performance by consuming large quantities of completely plant-based meals.

The energy requirements used in this study (4587.5 kcal/day) surpassed reported intakes in rugby players. A systematic review that used 24 h recalls and food diaries of varying durations to estimate the dietary intakes of team sport athletes including rugby players reported mean energy intakes that ranged from 3177 to 4336 kcal/day [70]. Additional research using FFQ, 24 h recalls, and food diaries estimated the energy intakes of rugby players to be between 2284 and 3875 kcal/day [8,10,22,23,71,72]. Low reported energy intakes relative to TEE have led to observations of a negative energy balance (−1382 kcal/day) in rugby players [10], accompanied by suggestions for cautious interpretation due to under-reporting during dietary assessment and limitations in energy intake estimations [10,72]. This study calculated nutrient values using a calorie level of 4587.5 kcal/day because this value precisely matches the mean TEE in rugby athletes determined using doubly labeled water [52]. Calculating nutrient intakes using a lower caloric value that was estimated from dietary assessments that are prone to significant measurement limitations [10,72] would therefore model a caloric deficit. A chronic negative calorie balance would limit the potential application of the results to rugby athletes because research suggests that, longitudinally, rugby players experience a small caloric surplus, as reflected by modest increases in total and lean mass over a seven-year period [73].

Modeled protein levels were 1.68 g/kg/day, which meet recommendations (1.6 g/kg/day) for rugby players [7,22,54,56,57]. Greater intakes have not been shown to further augment muscle mass or strength [74]. Rather, limited research suggests that moderate protein intakes may support muscular development in rugby athletes to a greater extent than higher intake levels [75], perhaps through the larger provision of dietary carbohydrates, which are known to promote glycogen storage and support exercise performance [76]. An interventional trial assigned 32 semi-professional rugby players to one of two isocaloric diets (3500 kcal/day) for six months: a Mediterranean-style diet providing 1.2 g/kg/day of protein and 6.1 g/kg/day of carbohydrate or a higher-protein diet providing 2.3 g/kg/day of protein and 5.5 g/kg/day of carbohydrate, each providing 30% of calories from fat [75]. Anthropometric changes were assessed using an electronic scale and skinfold measurements taken at four sites. Rugby players following the Mediterranean-style diet and higher-protein diet gained 4.9 and 1.2 kg of muscle mass, respectively (*p* = 0.03) [75]. These protein intakes and the values modeled in the present study reside within the range reported in surveys of the dietary intakes of rugby players, which indicate mean protein intakes of 1.02–2.59 g/kg [8,10,22,23,72,77]. These values may reflect larger intakes of protein supplements, the use of which is prevalent among rugby players [71,78,79,80]. In contrast, dietary data modeled from the AHS-2 cohort included limited capture of meal replacement drinks and protein supplements, where the top reported sources of protein included legumes, grains, and other whole plant foods [81].

Meat and animal products commonly supply a larger proportion of dietary protein than plant-based protein sources in the diets of rugby athletes [9,10,71,82]. The dietary habits of 21 professional rugby league players were assessed using FFQ, and between 80 and 100% of rugby players reported consuming eggs and cheese occasionally or often, whereas only 40–64% of the sample reported consuming legumes with the same frequency [82]. The dietary habits of 24 semi-professional rugby players were also evaluated using FFQ, and large intakes of animal protein sources (865 g/day of meat, eggs, and dairy) relative to plant protein sources were reported (17 g/day of nuts and seeds; legume intake not reported) [71]. Furthermore, research using FFQ to assess the dietary habits of 35 elite Rugby Union players found that intakes of meat (14.1 servings/week versus 2–4 recommended servings) and eggs (6.59 servings/week versus 2–4 recommended servings) exceeded recommendations, although nuts were reportedly under-consumed (1.19 servings/week versus 3–7 recommended servings) [10]. Meat and animal products may therefore displace nutrient-rich plant foods in the diets of rugby players, where low intakes of fruits, vegetables, whole grains, and nuts have been reported [9,10,22,23,82]. Recommendations have accordingly been made for rugby players to increase their consumption of fruits, vegetables, whole grains, legumes, and nuts [10,22,23].

The outcomes of leucine modeling indicate levels of 11.7 g/day, or 2.9 g/meal at each of four daily meals, which meet targets. These results align with findings from the Oxford arm of the European Prospective Investigation into Cancer and Nutrition (EPIC–Oxford), where young men adhering to completely plant-based diets were reported to consume a comparable percentage of calories from leucine [83]. Extrapolating these levels to those in this modeling study would yield leucine values of 10.9 g/day or 2.7 g/meal at four daily meals. These doses are substantial enough that additional leucine is not anticipated to result in further gains in muscle mass and strength [84,85].

The modeled nutrient levels exceeded requirements established in the USA, England, Australia, and New Zealand for vitamins A, B6, B9, B12, C, and E, calcium, iron, magnesium, phosphorus, potassium, zinc, fiber, omega-3 fatty acids, and linoleic acid. Vitamin D targets were met according to national guidelines in Australia and New Zealand but did not meet recommendations in the USA or England. Previous dietary analyses of rugby players’ diets suggest that mean intakes in this population meet the recommendations for vitamins B3, B6, and B12, as well as iron, phosphorus, and selenium [10,22,23,71]. However, mean intakes of vitamins A and E, magnesium, and folate are reportedly inadequate in this population, and mean intakes of vitamins B1, B2, C, and D as well as calcium, potassium, and zinc inconsistently meet recommendations [10,22,23]. These results indicate that it can be challenging for rugby athletes to satisfy vitamin D recommendations, and that regular consumption of a supplemental source of vitamin D should be encouraged [86]. Free radical-mediated cellular stress plays a critical role in the adaptation of tissues to exercise training, and it has been proposed that the increased intake of antioxidant nutrients could interfere with these hormetic processes [87,88]. Nutritive antioxidants include vitamin C, vitamin E, and carotenoids [89], and levels of these nutrients exceeded requirements in this modeling study. Antioxidant supplementation has been shown to diminish the adaptive response to exercise training [90]. However, the intake of antioxidant nutrients through whole foods or their extracts, even at levels that greatly exceed requirements, has not been shown to mitigate cellular adaptations to exercise [91,92,93]. The fiber values calculated in this study (107 g/day) surpassed targets (64 g/day). This contrasts with fiber intakes reported in previous studies of rugby players, which were between 19 and 22 g/day [10,22,71] and did not meet recommended levels. This may be consequential because dietary fiber can influence satiety and potentially diminish total energy intake in athletes [38,41,45]. Consuming large amounts of dietary fiber in whole, plant foods, such as the 107 g/day modeled in this study, has been suggested to predispose athletes to low energy availability [94]. This can be avoided by implementing strategies to ensure the inclusion of calorie-dense plant foods such as dried fruit and nut butters [46]. Furthermore, it has been suggested that the high fiber content of vegetarian diets could theoretically elicit gastrointestinal symptoms in athletes [95]. However, it has been proposed that the gastrointestinal tracts of athletes undergo adaptations that allow for larger calorie intakes through mechanisms such as increased rates of gastric emptying and nutrient absorption and reduced bloating and perceptions of fullness with greater tolerance to larger volumes of food [96]. High-carbohydrate diets have also been suggested to augment sodium-dependent glucose-1 (SGLT1) transporter density and activity, facilitating glucose transport and enhancing carbohydrate absorption and oxidation during exercise [96]. The high carbohydrate content (62% of total calories) in the modeled diets of the AHS-2 cohort [50] may therefore enable greater substrate utilization in athletic populations. Nonetheless, it has been recommended that athletes at increased risk of gastrointestinal disturbances meet calorie needs without inducing digestive distress or undue satiety by eating solid meals in the hours preceding competition that are low in fat, high in carbohydrates, and include limited fiber contents (e.g., waffles or pancakes with fruit), or to consume liquid meals (e.g., smoothie made from fruit and soy milk) in greater proximity to competition [97]. The omega-3 levels modeled in this study (5 mg/day) exceeded requirements (1.6 mg/day), although previous research has found that male rugby athletes who do not supplement with omega-3 fatty acids report mean intakes of 0.94 mg/day, which do not satisfy requirements [98]. This may be particularly relevant to rugby players because omega-3 fatty acids have been discussed as potentially performance-enhancing nutrients in team-based athletes due to their capacity to reduce measures of inflammation and preserve muscle membrane integrity following strenuous exercise [99]. Sodium levels (8099 mg/day) surpassed the recommended upper limit (2000–2300 mg/day) [11]. This exceeds mean sodium intake levels reported in college club rugby players following omnivorous diets, which were calculated using three 24 h diet recalls to be 4294 mg/day [22]. However, AHS-2 used an FFQ to calculate dietary sodium, which may significantly overestimate intake [100]. Additionally, rugby players demonstrate high sodium sweat losses of 44.0 ± 12.1 mmol·L^−1^ [101] and may be at risk for electrolyte imbalances [102]. A cohort study observing professional Rugby Union players found that, following match play as well as field and gym training, 57% of athletes who hydrated sufficiently to maintain their pre-training body mass exhibited hyponatremia [103]. Dietary sodium limits for the general population may therefore require modification for athletes with significant sodium sweat losses, such as rugby players. Finally, saturated fat levels were 5% of total calories, which meets recommendations to maintain intakes below 10% of total calories [11]. This is significantly lower than saturated fat intakes reported in rugby players following omnivorous diets, which are reportedly 11–13% of total calories [10,22,54,71]. This difference in saturated fat values is consequential because reducing saturated fat intake to recommended levels is advised to reduce the risk of CVD [104], the leading cause of death among former rugby athletes [20].

This study has strengths. First, following similar methods to our previous modeling research in male bodybuilders and American football players [30,31], a large cohort following completely plant-based diets was used to derive dietary data [50]. Second, calorie requirements were obtained from a systematic review that reported the energy expenditure values of rugby players using doubly labeled water [52], the gold standard for measurement accuracy [53].

This study also has several limitations. First, dietary levels were determined through mathematical modeling rather than direct measurement, which could produce variation between predicted and observed intakes. Second, we were unable to account for the consumption of performance-enhancing nutrients, which may vary across dietary patterns, such as creatine, due to the lack of reported data in large cohorts following plant-based diets [105]. The absence of dietary creatine has been suggested to potentially restrict the anabolic capacity of plant-based diets [41,106,107,108,109]. Experimental evidence demonstrates that supplemental creatine enhances RET-induced muscle hypertrophy [110] and strength [111,112], but evidence to support the notion that dietary creatine produces these effects is lacking [113]. Third, rugby is an international sport, yet the dietary intakes used for modeling derive from an American cohort [50] and may not reflect cross-cultural food choices among athletes in other countries.

This study modeled dietary intakes in adult male rugby players following completely plant-based diets. Future research should model plant-based diets among female athletes who engage in sports in which levels of lean body mass predict athletic performance [114,115]. Observational research in which athletes are provided completely plant-based meals during periods of rigorous physical activity would also provide insights into feasibility and potential application, and it will be helpful to assess the degree to which more energy-dense plant foods (e.g., nuts, seeds, and dried fruit) are required to meet the elevated energy needs of athletes. Comparing the effects of completely plant-based diets versus omnivorous diets that meet dietary guidelines on biomarkers of CVD would also be valuable.

## 5. Conclusions

This modeling study demonstrated that completely plant-based diets that are scaled to the energy requirements of adult male rugby athletes achieve recommendations for protein and leucine. These results suggest that the contents of large meal portions of completely plant-based diets are compatible with maximal muscular development and athletic performance in rugby players. Recommended targets for essential nutrients, with the exceptions of vitamin D and sodium, were also achieved. These findings indicate a potential convergence of sports nutrition recommendations for maximal muscle growth, strength, and athletic performance with dietary guidelines for chronic disease prevention and health promotion.

## Figures and Tables

**Table 1:** Calorie requirements and modeled protein and leucine values in adult male rugby athletes adhering to completely plant-based diets.

Body Mass (kg) [52]	Energy Requirements (kcal/day) [52]	Absolute Protein Requirements (g/day) [55]	Modeled Absolute Protein Levels (g/day)	Modeled Relative Protein Levels (g/kg/day)	Modeled Absolute Leucine Levels (g/day) [59]	Modeled Leucine Levels per Meal (g)
98.9	4587.5	158	166	1.68	11.7	2.9

g: grams; kcal: calories; kg: kilograms.

**Table 2:** Modeled micronutrient and other nutrient levels compared to requirements in adult male rugby players following completely plant-based diets.

	AHS-2 Strict Vegetarians *	Male Rugby Players	Nutrient Target in USA (Source) †	Nutrient Target in England (Source) ‡	Nutrient Target in Australia and New Zealand (Source) §	Targets Met?
Calorie Intake (kcal)	2000	4587.5	-	-		N/A
Saturated Fat						
(g)	25	25		<31		✓
(% of kcal)	5	5	<10 (DGA)	<10%	<10%	✓
Omega-3						
(g)	2 ¶	5 ¶	1.6 # (AI)		1.3 # (AI)	✓
(% of kcal)	0.9	0.9		0.5 # (AI)		✓
Linoleic Acid						
(g)	20	45	17 (AI)		13 (AI)	✓
(% of kcal)	9	9		4 (AI)		✓
Fiber (g)	47	107	64 ** (DGA)	25 (AI))	30 (AI)	✓
Vitamin A (mcg RAE ††)	1108	2541	900 (RDA)	750 (PRI)	900 (RDI)	✓
Vitamin B6 (mg)	14.4	33	1.3 (RDA)	1.7 (PRI)	1.3 (RDI)	✓
Folate (mcg)	888	2037	400 (RDA)	330 (PRI)	400 (RDI)	✓
Vitamin B12 (mcg)	23.3	53.4	2.4 (RDA)	4.0 (AI)	2.4 (RDI)	✓
Vitamin C (mg)	531	1218	90 (RDA)	119 (PRI)	45 (RDI)	✓
Vitamin D (IU)	252	578	600 (RDA)	600 (AI)	200 (AI)	✗
Vitamin E (mg)	101	232	15 (RDA)	13 (AI)	10 (AI)	✓
Calcium (mg)	1156	2652	1000 (RDA)	1000 (PRI)	1000 (RDI)	✓
Iron (mg)	32	72	8 (RDA)	11 (PRI)	8 (RDI)	✓
Magnesium (mg)	652	1496	400 (RDA)	350 (AI)	400 (RDI)	✓
Phosphorus (mg)	1371	3145	700 (RDA)	550 (AI)	1000 (RDI)	✓
Potassium (mg)	4234	9712	3400 (AI)	3500 (AI)	3800 (AI)	✓
Sodium (mg)	3531	8099	2300 (CDRR)	2000 mg (AI)	2000 (SDT)	✗
Zinc (mg)	16	37	11 (RDA)	16.3 (PRI)	14 (RDI)	✓

AI: adequate intake; AHS-2: Adventist Health Study 2; AR: average requirement; BM: body mass; CDRR: Chronic Disease Risk Reduction Level; DGA: Dietary Guidelines for Americans; N/A: not applicable; PRI: Population Reference Intake; RAE: Retinol Activity Equivalent; RDA: Recommended Dietary Allowance; RDI: Recommended Daily Intake; SDT: Suggested Daily Target. * Calorie intake represents the estimated energy requirements needed to maximize muscle mass and strength for athletic performance in adult male rugby players. Average micronutrient intakes per 2000 kcal/day reported in the AHS-2 dataset [50] were then scaled to meet these levels. † Nutrient targets sourced from the United States Department of Agriculture Dietary Guidelines for Americans 2020–2025 [11]. ‡ Nutrient targets sourced from the European Food Safety Authority and Public Health England [61,62,64]. § Nutrient targets sourced from the Australian Government Department of Health and Ageing, National Health and Medical Research Council [63,65]. ¶ Omega-3 intakes in AHS-2 and modeled levels for rugby players are for total omega-3 fatty acids. # Omega-3 intake recommendations are for alpha-linolenic acid. ** Based on recommendations to consume 14 g/1000 kcal. †† RDA for vitamin A provided in RAE to account for different bioactivities of provitamin A carotenoids [66]; ✓: target met; ✗: target not met.

## Data Availability

The original contributions presented in the study are included in the article.
